# IS EDUCATION ENOUGH? SEATED YOGA AND FALLS PREVENTION FOR OLDER ADULTS

**DOI:** 10.1093/geroni/igad104.3750

**Published:** 2023-12-21

**Authors:** Betsy Kemeny, Heather Bright, Leanne Digman, Krista McCormack, Riley Page, Madison Setto

**Affiliations:** Slippery Rock University, Slippery Rock, Pennsylvania, United States; Slippery Rock University, Slippery Rock, Pennsylvania, United States; Slippery Rock University, Slippery Rock, Pennsylvania, United States; Slippery Rock University, Slippery Rock, Pennsylvania, United States; Slippery Rock University, Slippery Rock, Pennsylvania, United States; Slippery Rock University, Slippery Rock, Pennsylvania, United States

## Abstract

Falls impact 25% of older adults 65+ annually (Bergen et al., 2016) with 1 in 5 falls resulting in trauma (CDC, 2020). Standing yoga can reduce falls by 48% (Hamrick et al., 2017), but some older adults cannot participate in these poses/inversions due to pain, lack of balance, and endurance. Seated yoga can reduce pain, fear of falling, mobility, and balance ( Yao & Tseng, 2019). Ong et al. (2021) found that falls education improves intention to engage in prevention. Although CDC (2022) suggests multi-modal interventions , no known research compares a combination of falls prevention education and seated yoga intervention with an education-only control group. This controlled study compared fifteen older adults living in HUD housing who received both an 8-week “Get Fit While You Sit” seated yoga (LVSY, 2021) and falls prevention education to those who only received falls prevention education. Physical testing (Timed Up and Go (TUG), chair stand test, 4-point balance test) and attitude/participation measures were measured in both groups. Wilcoxon signed rank and Kruskal-Wallis were used to compare pre-to-post and between groups. Results suggest that the multi-modal intervention (combined education and chair yoga) resulted in improvements in TUG and chair stand test but other measures (e.g. self-efficacy for falls) did not change over time. The education-alone group did not improve over time. Between groups, a sig. difference was found in TUG and Chair stand. More research is needed but older adults in LTC settings may benefit from combining seated yoga with prevention education.

